# A Combinatorial Algorithm for Microbial Consortia Synthetic Design

**DOI:** 10.1038/srep29182

**Published:** 2016-07-04

**Authors:** Alice Julien-Laferrière, Laurent Bulteau, Delphine Parrot, Alberto Marchetti-Spaccamela, Leen Stougie, Susana Vinga, Arnaud Mary, Marie-France Sagot

**Affiliations:** 1 Erable team, INRIA Grenoble Rhône-Alpes, 655 avenue de I’Europe, 38330 Montbonnot-Saint-Martin, France; 2 University Lyon 1, CNRS UMR 5558, F-69622 Villeurbanne, France; 3 Université Paris-Est, LIGM (UMR 8049), CNRS, UPEM, ESIEE Paris, ENPC, F-77454, Marne-la-Vallée, France; 4 Sapienza University of Rome, Italy; 5 VU University and CWI, Amsterdam, The Netherlands; 6 IDMEC, Instituto Superior Técnico, Universidade de Lisboa, 1049-001 Lisboa, Portugal

## Abstract

Synthetic biology has boomed since the early 2000s when it started being shown that it was possible to efficiently synthetize compounds of interest in a much more rapid and effective way by using other organisms than those naturally producing them. However, to thus engineer a single organism, often a microbe, to optimise one or a collection of metabolic tasks may lead to difficulties when attempting to obtain a production system that is efficient, or to avoid toxic effects for the recruited microorganism. The idea of using instead a microbial consortium has thus started being developed in the last decade. This was motivated by the fact that such consortia may perform more complicated functions than could single populations and be more robust to environmental fluctuations. Success is however not always guaranteed. In particular, establishing which consortium is best for the production of a given compound or set thereof remains a great challenge. This is the problem we address in this paper. We thus introduce an initial model and a method that enable to propose a consortium to synthetically produce compounds that are either exogenous to it, or are endogenous but where interaction among the species in the consortium could improve the production line.

Synthetic biology has been defined by the European Commission as “the application of science, technology, and engineering to facilitate and accelerate the design, manufacture, and/or modification of genetic materials in living organisms to alter living or nonliving materials”. It is a field that has boomed since the early 2000s when in particular Jay Keasling showed that it was possible to efficiently synthetise a compound–artemisinic acid–which after a few more tricks then leads to an effective anti-malaria drug, artemisinin^1^. Such chemical compounds were naturally produced only in the plant *Artemisia annua*, a type of wormwood, in quantities too small to enable a cheap production of the drug. To address this problem, a living organism, *Saccharomyces cerevisiae*, was used for such rapid, and therefore much more effective synthetic production. Since the work by J. Keasling, many other species, in particular bacteria, have also been manipulated with a similar objective of more efficiently producing some compounds of interest for health, environmental or industrial purposes.

However, engineering a single microorganism to optimise one or a collection of metabolic tasks may often lead to considerable difficulties in terms either of getting an efficient production system, or of avoiding toxic effects for the recruited microorganism^2^. The idea of using a microbial consortium has thus started being developed in the last decade^2^,^3^,^4^,^5^. This can indeed allow to perform more complex tasks, for example by splitting the work between the members of the consortium, by alleviating an inhibition due to toxic compounds as we show later, or even by obtaining a culture more resistant to environmental changes. Microorganisms may thus be more efficient synthetic factory workers as a group than as individual species, as already shown for problems related to remediation or energy^6^,^7^. However, difficulties may arise, limiting or preventing the success of such community approaches^8^,^9^. Finally, selecting the members of the consortium to produce one or several compounds remains a challenge^2^.

In this paper, two types of consortia are studied. The first is a synthetic consortium of strains carrying genetic and/or regulatory modifications. This follows the same spirit as in the work of Jay Keasling for the production of artemisinic acid. In our case, the goal is the synthetic production of two bioactive compounds with antibacterial properties: penicillin and cephalosporin C. Four microorganisms were considered for such production. Notice that already an important question is whether the best option is to use all four in the consortium, or instead only a subset thereof, and of course, which subset is then most efficient. In this first case study, the compounds of interest are exogenous to the consortium.

In the second case study addressed, the two microorganisms form an artificial consortium in the sense that the species involved in it do not naturally interact, and both organisms are able to endogenously produce the target compounds. One of these is 1,3-propanediol (PDO), a building block of polymers. Associating microorganisms in a consortium can lead to a better yield of production as already demonstrated by Bizukojc *et al*.^10^. This however is not the only consortium that may be considered.

In both cases, it is necessary to infer the transfer of metabolites from one organism to another and, if the compounds are exogenous to the selected organisms, which reactions need to be inserted in the consortium. For such problems, computer models are crucial in providing hints on how to best divide a given metabolic production line among different organisms that are then made to interact with one another. Various methods exist that enable to better understand the metabolic capabilities and the interactions observed in natural communities^11^,^12^, but they do not take into consideration the production of specific products from selected substrates. This issue was addressed more recently by Eng and Borenstein^13^ while minimising the number of species in the community. In this paper, we present a different model to solve both biological cases considered above that attempts to strike a balance between the exchanges that would be required among the species involved in the consortium and the genetic modifications that would be needed. To this purpose, we use a weighted network, thus assigning a priority of use to some reactions over others. This enables on one hand to either favour or on the contrary, disfavour a transport reaction, and on the other hand to reflect the difficulties associated with inserting exogenous genes.

Indeed, the problem of obtaining an optimal consortium includes at least the following two parallel objectives: one is to have a small number of reactions exogenous to the consortium that need to be added to it, the second is to have a small number of compounds that need to be transported across different species of the consortium. Both are indeed costly and should thus be avoided whenever possible. Other aspects would also need to be taken into consideration, such as the efficiency of the consortium in terms of both survival and growth of each species composing it, as well as of production of the compounds of interest. In this paper, we address only the first two objectives of minimising the number of insertions of exogenous reactions and of transitions. Our approach is purely combinatorial and topological. We do not take into account stoichiometry for the moment. This approach however represents a first step that, as we show, leads already to a hard problem. We start by some preliminaries that present the basic notations and definitions used, the model adopted, and a formal description of the problem addressed. Following the idea initially introduced by Fellows *et al*.^14^,^15^, we then explore how different parameters of the problem and combinations thereof influence its complexity. We propose an initial algorithm, MultiPus, for addressing this problem. However, because of an increasing running time on genome-scale metabolic models (GEMs), MultiPus is also available using an *Answer Set Programming* (ASP) solver^16^ which is more efficient in general. Finally, we present the two production cases explored with MultiPus.

## Preliminaries

### Notations and basic definitions

We work with a directed hypergraph representation of a metabolic network, using genome-scale metabolic models (GEMs). Let then 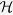 be a directed hypergraph defined on a set of vertices, denoted by *V*, that corresponds to the compounds, and a set of directed hyperedges, that is of *hyperarcs*, denoted by *A*, that corresponds to the reactions. Given a hyperarc *a*, we denote by src(*a*) and tgt(*a*) the sets of source and target vertices of *a*, respectively, that is the set of substrates and of products. In the problem described below, the main issue comes from the hyperarcs with multiple source vertices. The possible multiplicity of the target vertices of a hyperarc does not affect the complexity of the problem. Moreover, we can, without loss of information, decompose such hyperarcs into ones that each have the same set of source vertices but only one of the target vertices of the original hyperarc (as explained in the Supplementary Material). We therefore make this assumption from now on.

For a subset of hyperarcs *A*′ ⊆ *A*, *V*(*A*′) denotes the set of vertices that are involved in at least one hyperarc of *A*′, that is the set of compounds that participate in at least one of the reactions represented by *A*′. By abuse of notation, given a set of hyperarcs *A*′, we often refer to the hypergraph (*V*(*A*′), *A*′) simply as *A*′.

Since a reaction needs all its substrates to be activated, we consider that the multiple source vertices of a hyperarc correspond to a multiplicity of tentacles (often used for grasping), each associated to one substrate. A hyperarc is therefore like an octopus, only with a number of tentacles that may be different from eight. The greater the number of tentacles, the more tentacular is the hyperarc considered to be.

We formally introduce the notion of a *tentacular* hyperarc as follows.

**Definition 1.** *A hyperarc a is called* tentacular *with* number of tentacles*, or* spreadness *for short, b if b = |*src*(a)| > 1.*

Finally, we define the notion of the *total number of tentacles*, *total spreadness* for short, of a directed hypergraph.

**Definition 2.** *Given a directed hypergraph H(V,A), its total spreadness is the sum of the number of sources of the tentacular hyperacs in* 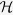.

For the sake of simplicity, we will use the term *arc* to refer to non tentacular hyperarcs. It will later become clear why we need to consider the total spreadness of the input.

### Model adopted

We recall that the problem we want to address concerns the production by a consortium of organisms, microbes for instance, of a set of compounds denoted by *T*. The compounds of interest may not be produced naturally by the members of the consortium; they are instead produced by other organisms (in the example given in the introduction, this is a plant). We denote these two sets by, respectively, *O*_*w*_ (the workers to be used to synthetically produce the compounds in *X*) and *O*_*o*_ (those other organisms, used as reference, where the compounds in *T* are naturally produced). As indicated, we may have |*O*_*o*_| = 0 meaning here that the workers are naturally able to produce the compounds.

Let *N*_1_, …, *N*_*k*_ be the genome-scale metabolic models (GEMs) for the organisms in *O*_*w*_, and let *V*_1_, …, *V*_*k*_ respectively correspond to the sets of vertices in these networks. Actually, this is a superset of the consortium that may really be required for the production of *T* and that will be a solution of the problem as defined below. The hyperarcs in *N*_*i*_ have weight *w*_*worker*_, independently of *i*.

Typically *w*_*worker*_ will be set equal to zero, or to a value that is close to zero for reasons that will be explained later, in the Application part. The set of hyperarcs in the metabolic models for *O*_*o*_ is denoted by *A*_*o*_.

The directed hypergraph 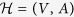 that is the input to our problem is constructed in the following way.

First, we perform the disjoint union of the networks *N*_1_, …, *N*_*k*_. Let 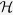 be such that 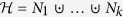. Thus for now 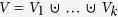 and 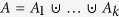. Then, for each network *N*_*i*_, and for each hyperarc *a* ∈ *A*_*o*_ that corresponds to a reaction not already in *N*_*i*_, we create a copy of it in *N*_*i*_, and thus in 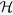. We add the hyperarc *a* labelled as *a*_*i*_ to *A*_*i*_. We further add to *V*_*i*_, and thus to *V* any vertex corresponding to a compound not already in *N*_*i*_ if such exists. The added hyperarc has weight *w*_*other*_. Typically, *w*_*other*_ > *w*_*worker*_: introducing a reaction in the metabolism of an organism that does not contain the corresponding enzyme(s) is indeed costly. Finally, for each pair of vertices *v*_*i*_ ∈ *V*_*i*_ and *v*_*j*_ ∈ *V*_*j*_ with *i*, *j* ≤ *k* and *i* ≠ *j* such that the corresponding compound is the same, we create a hyperarc that has *v*_*i*_ for single source and *v*_*j*_ for single target (it therefore is an arc) and has weight *w*_*transition*_. Typically, we will have that *w*_*transition*_ > *w*_*worker*_: making a transition from one organism of the synthetic consortium to another, which implies transporting a compound, is also costly.

It is worth calling attention to the fact that we are considering here that adding a reaction from *O*_*o*_ to an organism from the consortium *O*_*w*_ (when such operation is required) implies a cost that does not depend on the reaction. Similarly, we are considering that any transition from one organism in *O*_*w*_ to another is equally costly. These assumptions may however be refined by making such costs, and thus the weights of the added hyperarcs (tentacular hyperarcs or arcs) depend on the reaction or on the transition (see later for a further discussion on this).

### Problem definition

We first introduce the notion of a directed rooted hypergraph.

**Definition 3.** *A directed hypergraph* 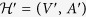 *is rooted at S ⊆ V′ if there exists an ordering of its hyperarcs (a*_*1*_*, …, a*_*m*_*) such that for all i ≤ m*, src*(a*_*i*_*) ⊆ S ∪ * tgt*({a*_*1*_*, …, a*_*i−1*_*}).*

The problem that we address in this paper is defined as follows:

### Directed Steiner Hypertree (DSH) problem

**Input:** A weighted directed hypergraph 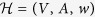 where w is the set of weights associated to the hyperarcs in A, a set of sources S and a set of targets T.

**Output:** A directed hypergraph 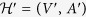 rooted at S, with V′ ⊆ V and A′ ⊆ A, of minimum weight such that T ⊆ tgt(A′).

Notice that the term Directed Steiner Hypertree abuses language in the sense that there may be more than one root. In the case of digraphs, it would correspond to a set of trees, hence to a forest.

### Relation to known problems

If the directed hypergraph is a digraph, then it is a minimal directed hypergraph rooted at a node *s* if and only if it is an arborescence rooted at *s* (*i.e.* a directed tree with an orientation from the root *s* to the leaves). If there is more than one source, then it is a set of arborescences. In the case of digraphs, the DSH problem coincides with the well-studied Directed Steiner Tree (DST) problem defined as follows:

### Directed Steiner Tree (DST) problem

**Input:** A weighted directed graph G = (V, A), a source s and a set of targets T.

**Output:** A subset A′ of A of minimum weight such that T ⊆ closure_A′_(s).

The closure operation is defined as follows: Given a directed hypergraph 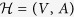, a set of vertices *X* such that *X* ⊆ *V* and a set of hyperarcs *A*′ such that *A*′ ⊆ *A*, closure_*A*′_(*X*) is the smallest set *C* ⊆ *V* such that *X* ⊆ *C* and for each *a* ∈ *A*′, if src(*a*) ⊆ *C*, then tgt(*a*) ⊆ *C*.

Intuitively, closure_*A*′_(*X*) is the set of vertices that can be reached from *X* following the hyperarcs in *A*′. In the context of metabolic networks, it is the set of compounds that the reactions from *A*′ can produce using only the compounds of *X* as sources.

### Complexity of the problem

We start by investigating the complexity of the problem. We first observe that the Directed Steiner Tree problem is NP-hard^17^. The Directed Steiner Hypertree problem is also NP-hard, even on graphs, indicating that it is highly unlikely that there exists an efficient (polynomial time) delay algorithm for its solution. However, if the number of targets is considered a constant, then there exists an algorithm with polynomial running time. DST is said to be Fixed Parameter Tractable (FPT) with the number of targets as parameter. This implies that DSH also admits an FPT algorithm for a constant number of targets in the case where the input is a directed graph.

In the general setting however, Proposition 1 indicates that the problem is doomed to be intractable when using only parameters related to the solution size. The proofs of the propositions are available in the Supplementary Material and in the Supplementary Figures S1 and S2.

**Proposition 1.** *The problem is W[1]-hard when parameterised by any combination of: |A′|, weight(A′), |T|, |S|, total number of tentacles of the hyperarcs in A′.*

Part of the difficulty indeed comes from the choice of tentacular hyperarcs that must belong to the solution. However, taking into account only the number of tentacular hyperarcs in the instance is not sufficient to obtain tractability.

**Proposition 2.** *The problem is NP-hard even when |T| = 1 and A contains only one tentacular hyperarc.*

Overall, the problem remains intractable when either of these constraints applies to the input: there are few targets, or the total number of tentacles of the hyperarcs is bounded. However, there remains the stronger case when both quantities (number of targets and total number of tentacles of the hyperarcs) are bounded. We present a fixed-parameter tractable algorithm for this case in the next section.

## Algorithm

We now present our main algorithm that exactly solves the Directed Steiner Hypertree problem provided that the number of targets and the total number of tentacles of the hyperarcs remain small. Intuitively speaking, the algorithm identifies the best combinations of tentacular hyperarcs by trying all those in parallel, and for each such combination, it outputs the solutions (if any exists) having minimum weight. More precisely the algorithm enumerates all possible combinations of tentacular hyperarcs that will be used in a solution, where a combination is a subset of the tentacular hyperarcs ordered according to the topological order of the solution (with *k* tentacular hyperarcs, there are 2^*k*^*k*! such combinations to consider). For each combination, it remains to compute the optimal way of linking these tentacular hyperarcs with regular arcs. This problem is solved by extending the FPT algorithm for the Directed Steiner Tree problem which requires the number of targets as a parameter. In our case, we need the number of targets plus the total number of tentacles of a solution. For a given directed weighted hypergraph 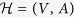, we denote by 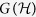 the graph obtained from 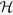 by removing all tentacular hyperarcs. Let ST(*x*, *X*) be the best directed Steiner tree of 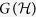 rooted in *x* that has *X* as set of leaves.

Given an ordered subset *M* := (*a*_1_, …, *a*_*k*_) of the tentacular hyperacs of 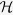, we describe a dynamic programming algorithm to find the best Directed Steiner Hypertree with hyperarc set *A*′ that uses exactly the tentacular hyperarcs of *M* following their ordering.

**Figure 1:**
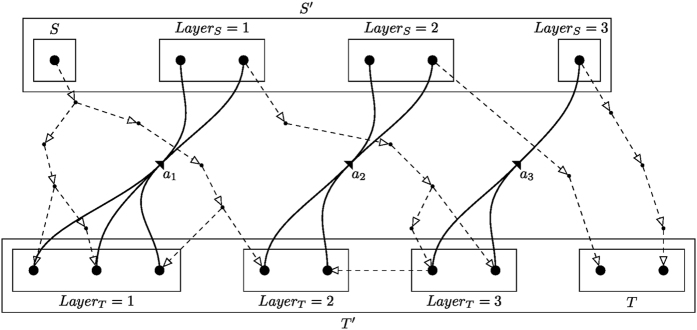
Illustration of the notion of layers: Given *M* = (*a*_1_, *a*_2_, *a*_3_) (thick tentacular hyperarcs), and a solution *A*′ containing *M*, *G*(*A*′) (dashed arcs) consists of a forest covering all *T*′, *i.e.* each vertex in *t* ∈ *T*′ is part of a tree whose source is in a lower “layer” than *t*.

The following definitions are illustrated in Fig. 1. Since all tentacular hyperarcs of *M* must be used, we have that, for all *i* ≤ *k*, src(*a*_*i*_) ⊆ tgt(*A*′) ∪ *S*, and so the set src(*M*) can be seen as an additional set of targets. We establish *T*′ := *T* ∪ src(*M*) to be the new set of targets, and for *t* ∈ *T*′, we define *Layer*_*T*_(*t*) := min{*i* ≤ *k*: *t* ∈ src(*a*_*i*_)}. If *t* ∈ *T*\src(*M*), we define *Layer*_*T*_(*t*) as *k* + 1, and for a subset *X* ⊆ *T*′, we define *Layer*_*T*_(*X*) := min{*Layer*_*T*_(*t*): *t* ∈ *X*}. Similarly, since all tentacular hyperarcs of *M* must be used, intuitively tgt(*M*) can be seen as an additional set of sources. We write *S*′ := *S* ∪ tgt(*M*) and *Layer*_*S*_(*s*) := min{*i* ≤ *k*: *s* ∈ tgt(*a*_*i*_)} if *s* ∈ tgt(*M*)\*S*, and *Layer*_*S*_(*s*) := 0 if *s* ∈ *S*. To respect the ordering of *M*, the target of a tentacular hyperarc *a*_*i*_ ∈ *M* can be used to “reach” only the sources of the tentacular hyperarcs that come after *a*_*i*_ in *M*. For all *Y* ⊆ *T*′, we define *S*_*Y*_ := {*s* ∈ *S*′|*Layer*_*S*_(*s*) < *Layer*_*T*_(*Y*)}.

Observe that for any minimal Directed Steiner Hypertree *A*′, the vertices in *G*(*A*′) must have in-degree one if they are not in *S*′, and, by minimality, out-degree at least one if they are not in *T*′.

Given a (directed) forest *F*, we denote by *V*(*F*) and leaves(*F*) respectively the vertices and the leaves of all the trees of *F*. For any vertex *t*, we denote by root(*F*, *t*) the root of the tree in *F* containing *t* when *t* ∈ *V*(*F*) (the root is the farthest vertex we can reach starting from *t* by following only branches of *F*), or root(*F*, *t*) = *t* otherwise (*t* is an isolated node).

For a set of targets *Y* ⊆ *T*′, we say that a forest *F* of 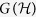 *covers Y* if leaves(*F*) ⊆ *Y* and root(*F*, *t*) ∈ *S*_*t*_ for all *t* ∈ *Y*.

**Lemma 1.** *For any optimal solution A′ of the Directed Steiner Hypertree problem given* 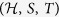 *as input, if A′ uses exactly the tentacular hyperarcs of an ordered subset M, then G(A′) is a forest covering T′.*

*Proof*. Consider a Directed Steiner Hypertree *A*′. First notice that by minimality, *G*(*A*′) is a forest. Indeed, if some vertex *x* has two incoming arcs in *A*′, denoted by *a* and *a*′, *a* appearing before *a*′ in *A*′, then removing arc *a*′ yields a strictly better solution to the Directed Steiner Hypertree problem. Furthermore, if any *x* ∉ *T*′ is a leaf of *G*(*A*′), with incoming arc *a*, then *x* is not the head of any arc nor is it part of *T*′. In this case, *a* can be deleted and all leaves are in *T*′.

Consider now any *t* ∈ *T*′. Let *s* = root(*F*, *t*). Consider the path from *s* to *t*: by minimality, the arcs of the path must appear in the same order as in *A*′ (otherwise some arcs must be deleted), and *s* must appear in the targets of a tentacular hyperarc ordered before any hyperarc of which *t* is a source (or *s* ∈ *S*). This implies that *s* ∈ *S*_*t*_. ◻

**Lemma 2.** *Given an ordered subset of tentacular hyperarcs M and any forest F covering T′, there exists a solution A′ of the Directed Steiner Hypertree problem with* 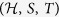 *as input, where A′ uses exactly the tentacular hyperarcs of M in this order, and such that G(A′) = F.*

*Proof.* We build *A*′ as follows. We first insert the tentacular hyperarcs (*a*_1_, …, *a*_*k*_) of *M*, in this order. We then insert the arcs of *F* between the tentacular hyperarcs, according to the layer of the root of the tree to which they belong. Formally, let *D*_1_, …, *D*_*p*_ be the directed trees in *F*, and *s*_1_, …, *s*_*p*_ their respective roots. Observe that since all leaves are in *T*′, then each *s*_*i*_ can be written as root(*F*, *t*) for some *t* ∈ *T*′, and thus *s*_*i*_ ∈ *S*′ and *Layer*_*S*_(*s*_*i*_) is well-defined and can be computed. For each *j*, 1 ≤ *j* < *k* (respectively, *j* = 0 or *j* = *k*), we insert between *a*_*j*_ and *a*_*j*+1_ (resp. before *a*_1_ or after *a*_*k*_), all arcs of all trees *D*_*i*_ such that *Layer*_*S*_(*s*_*i*_) = *j*. Within each tree, the arcs are inserted in topological order. There remains to prove that this ordering has the required properties.

We first verify that for any *t* ∈ *T*, *t* is reached by some hyperarc (tentacular or not) of *A*′. Two cases are possible:

1. If *t* = root(*F*, *t*) (*i.e.*, either *t* is the root of some tree of *F* or *t* ∉ *V*(*F*)), then *t* ∈ tgt(*a*_*i*_) for some *a*_*i*_ ∈ *M* ⊆ *A*′, thus *t* ∈ tgt(*A*′).
2. Otherwise, *t* ∈ tgt(*a*) for some arc *a* in *F*, so *a* ∈ *A*′ and *t* ∈ tgt(*A*′).

For any vertex *x* ∈ src(*a*) with *a* ∈ *A*′, we now need to verify that *x* ∈ *S* or *x* is the target of some hyperarc selected before *a*. Three cases apply:

1. If *a* ∈ *F* and *x* is not the root of any tree *D*_*i*_, then it has an incoming arc appearing in *A*′ before *a* (since we kept the topological order of each tree).
2. If *a* ∈ *F* and *x* is the root of some tree *D*_*i*_, then *x* = *s*_*i*_. If *x* ∉ *S*, then *Layer*_*S*_(*x*) > 0, and *x* is produced by the tentacular hyperarc 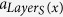 which appears before *a*.
3. If *a* is not an arc of *F*, then it is a tentacular hyperarc, *x* ∈ *T*′, and *a* = *a*_*j*_ for some *j* > *Layer*_*T*_(*x*). Let *s*_*i*_ = root(*F*, *x*), then *Layer*_*S*_(*s*_*i*_) + 1 ≤ *Layer*_*T*_(*x*), and the arc producing *x* is placed before 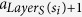, which in turn is before (or equal to) the arcs 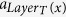 and *a* = *a*_*j*_.

Overall, we indeed have a Directed Steiner Hypertree for 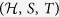 using *M*, where, by construction, *G*(*A*′) = *F*.◻

**Lemma 3.** *For any optimal solution A′ of Directed Steiner Hypertree of* 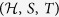
*, if A′ uses exactly the tentacular hyperarcs of an ordered subset of tentacular hyperarcs M in this order, then G(A′) is a forest covering T′ of minimum weight.*

*Proof.* By Lemma 1, *F* = *G*(*A*′) is already a forest, and it has a total weight of *weight*(*F*) = *weight*(*A*′) − *weight*(*M*). Consider any forest *F*′ of weight *w*′ covering *T*′. By Lemma 2, there exists a solution with weight *weight*(*F*′) + *weight*(*M*), which must be larger than *weight*(*A*′), hence *w*′ ≥ *weight*(*F*), *i.e. F* has minimal weight. ◻

For a set of targets *Y* ⊆ *T*′, let SH_*M*_(*Y*) be the minimum weight of a forest *F* covering *Y* under the ordering *M*. By Lemma 3, the weight of an optimal solution *A*′ of the Directed Steiner Hypertree problem given 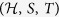 as input is *weight*(*M*) + SH_*M*_(*T*′) where *M* is the ordered set of tentacular hyperarcs used by *A*′.

**Theorem 1.** *The optimal value of an instance of* 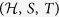 *of the Directed Steiner Hypertree problem has value SH*_*M*_(T′) + weight(M) for some ordering M. Furthermore, *SH_*M*_ *can be computed recursively as follows. For any Y ⊆ T′*,


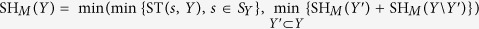


*Proof.* Assume first that the optimal forest *F* covering *Y* is a tree and let *s* ∈ *S*_*Y*_ be its root. Then SH_*M*_(*Y*) = ST(*s*, *Y*) = min{ST(*s*, *Y*), *s* ∈ *S*_*Y*_}.

Assume now that *F* has at least two trees. Let *Y*′ :=  leaves (*F*_1_) where *F*_1_ is a tree of *F*. Notice that since *F*_1_ and the other trees of *F* do not intersect, we have *weight*(*F*) = *weight*(*F*_1_) + *weight*(*F*\*F*_1_). Furthermore, *F*_1_ is an optimal forest covering *Y*′ and *F*\*F*_1_ is an optimal forest covering *Y*\*Y*′ since otherwise, the union of two better solutions would lead to a better forest covering *Y*. We then have that SH_*M*_(*Y*) = SH_*M*_(*Y*′) + SH_*M*_(*Y*\*Y*′) and 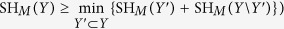. Finally, assume that there exists *Y*′ ⊆ *Y* such that SH_*M*_(*Y*) > SH_*M*_(*Y*′) + SH_*M*_(*Y*\*Y*′) and let *F*′ (resp. *F*′′) be an optimal forest covering *Y*′ (resp. *Y*\*Y*′). Then *F*′ ∪ *F*′′ would be forest covering *Y* of weight *weight*(*F*′ ∪ *F*′′) ≤ SH_*M*_(*Y*′) + SH_*M*_(*Y*\*Y*′) < *F*, contradicting the optimality of *F*. Thus 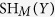

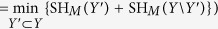
.◻

**Theorem 2.** *The Directed Steiner hypertree problem is Fixed-Parameter Tractable for the parameters |T| and total number of tentacles of the hypergraph.*

*Proof.* The algorithm computes SH_*M*_(*T*′) for each ordered subset *M* of tentacular hyperarcs. Since the number of tentacular hyperarcs is bounded by the total number of tentacles *k* of the hypergraph, there are at most 2^*k*^*k*! ordered subsets of tentacular hyperarcs. For a given *M*, we now compute SH_*M*_(*T*′) using a dynamic programming algorithm induced by the recursion of Theorem 1. We need to store the value of SH_*M*_(*Y*′) for every subset *Y*′ of *T*′. Since the size of *T*′ is bounded by *k* + |*T*|, we have at most 2^*k*+|*T*|^ such subsets. Finally, since for every vertex *s* and every *Y*′ ⊆ *T*′, the computation of ST(*s*, *Y*′) is FPT in |*Y*′| ≤ |*T*′| ≤ *k* + |*T*|, the total running time of the algorithm is FPT in *k* + |*T*′|.◻

## Application

The main objective of microbial consortia engineering is to highlight their capacity to reach enhanced productivity, stability or metabolic functionality^3^. More in particular in this paper, we explore the possibility of such consortia to produce compounds of interest using low cost substrates (such as, for instance, the waste of other industries).

We initially focused attention on the production of two bioactive compounds: penicillin and cephalosporin C, useful to the pharmacology industry for their antibiotic properties. For this production, a synthetic consortium defined as a system of metabolically engineered microbes which are modified by genetic manipulations and/or regulatory processes^2^ has been tested, using distant species as will be explained in the first example. The goal in this case was to take advantage of the different metabolic capabilities of the organisms composing the consortium for the *de novo* synthesis of bioactive metabolites and to show that the model is able to select the Directed Steiner Hypertree of least cost to produce one or a set of metabolites of interest.

We then considered the case of an artificial consortium. This corresponds to a system composed of wild-type populations that do not naturally interact^2^. We tested the association of a natural 1,3-propanediol (PDO) producer *Klebsiella pneumoniae* with an acetogenic Archae *Methanosarcina mazei*. The goal is to obtain a higher yield of 1,3-propanediol. Indeed, production of this compound in a pure culture of *K. pneumoniae* is associated with production of acetate. The latter has an inhibiting effect on bacterial growth, and ultimately also on the production of PDO. Hence associating *K. pneumoniae* with a methanogen has been proposed to reduce such effect^5^,^10^.

All the genome-scale models (GEMs) used were extracted from Kegg^18^ using MetExplore^19^. In both examples, cofactors and co-enzymes obtained from a list available in Kegg^18^ were removed. The networks, constructed as explained previously, were filtered using a lossless compression step (see Supplementary Material and Supplementary Figures S3 and S4). The resulting networks have a high number of tentacular hyperarcs. In the first case, the directed hypergraph contains 10087 arcs and 285 tentacular hyperarcs (that is, arcs with at least two substrates). The total number of tentacles of the graph is 575. In the case of improved PDO production, the network contains 1606 arcs and 71 tentacular hyperarcs for a total number of tentacles of 142. Because of the high number of total spreadness, we used an ASP (Answer Set Programming) solver^16^ to enumerate the optimal solutions, namely the sets of reactions with minimum total weight such that the target compound(s) could be produced using only the given substrate(s).

In the absence of any prior knowledge, the weights were set uniformly using as *a priori* the fact that endogenous reactions should be easier to use than transport ones (no need to export or to uptake compounds) and than insertions (since this implies introducing one or several genes and over-expressing them).

Therefore, the following weights were first applied: *w*_*worker*_ = 1, *w*_*other*_ = 100, *w*_*transition*_ = 100. Notice that the weight of the (hyper)arcs that are present in the organisms forming the consortium is not zero, but instead equal to a value above zero that remains however small in relation to the weights of an insertion or of a transition. The motivation for this is to favour solutions which, while minimising the number of insertion or transition hyperarcs that are used, also minimise the number of hyperarcs corresponding to reactions that are internal to the microorganisms in the consortium.

In the second application, two sets of transport weights were adopted, one a refinement of the first, as will be explained later on.

### Antibiotics production

In this first application, a synthetic consortium of three Actinobacteria (*Streptomyces cattleya*, *Rhodococcus jostii* RAH_1, *Rhodococcus erythropolis* BG43) and one methanogenic Archaea (*Methanosarcina barkeri*) was tested to determine which microbial consortium could produce a set of metabolites of interest. In this case, two well-known beta-lactam antibiotics (penicillin and cephalosporin C) were selected. Both active compounds belong to the cephalosporin/penicillin pathway and share several metabolic reactions. They also have a common precursor, namely isopenicillin N, are commonly used for their antibacterial properties and are naturally produced by fungi belonging to the *Aspergillus* and *Cephalosporium* species (*Aspergillus chrysogenum* and *Cephalosporium acremonium* respectively)^20^. In this case, cellulose was used as carbon source. Indeed, life on earth depends on photosynthesis, which results in the production of plant biomass having cellulose as major component, and cellulosic materials are particularly attractive in this context because of their relatively low cost and plentiful supply^21^.

Microorganisms were chosen because of the availability of Actinobacteria to produce bioactive compounds (representing about 45% of all the microbial bioactive products discovered^22^). Furthermore, the phylogenetic distance between Actinobacteria and Archaea suggests variability in their metabolisms. The presence of reactions that are specific to each organism means that there might be a gain in the overall metabolic capabilities from making the two bacteria work together. Using a consortium could thus be more efficient to produce one or several of the metabolites of interest. In addition, two other organisms (henceforth called *reference organisms*) were used for reaction insertion: *Aspergillus nidulans* and *Streptomyces rapamycinicus*. The first is a fungus known to produce penicillin while the second possesses reactions in the penicillin/cephalosporin C pathway, and in particular those needed to produce cephalosporin C. All the reactions present in the reference organisms were added to the four prokaryotes forming the consortium (as described in Model adopted).

**Figure 2:**
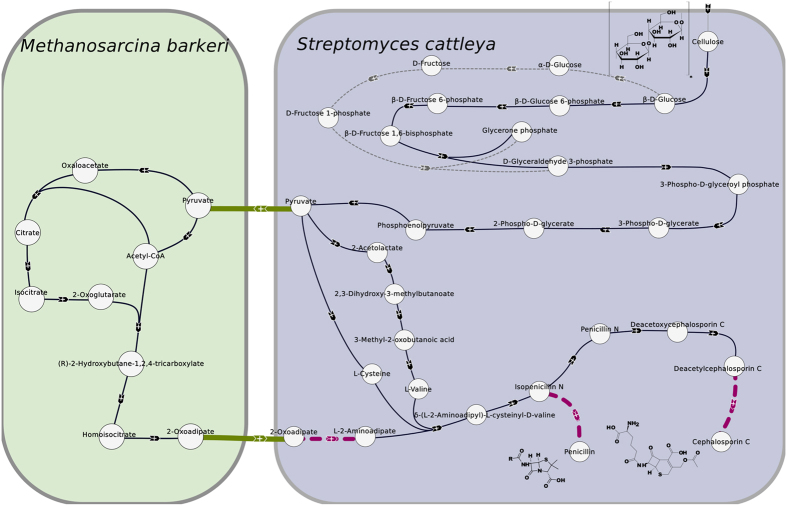
Representation of two solutions of minimum weights. The circles are compounds. Black hyperarcs are endogenous reactions, that is reactions already present in the organisms forming the consortium, while purple-dashed hyperarcs are the reactions that were inserted. Green arcs represent the transport of pyruvate from *Streptomyces cattleya* to *Methanosarcina barkeri* and of 2-oxoadipate from *M. barkeri* to *S. cattleya*. The widths of the arcs are proportional to the assigned weights. Grey-dashed arcs represent an alternative path of endogenous reactions in the upper part of glycolysis. Hence, the second solution uses this path instead of the one just below to link *β*-D-glucose to D-glyceraldehyde 3-phosphate.

Four solutions with a minimum cost of 528 (2 transports, 3 insertions, and 28 endogenous reactions) are found. All of them are composed of *Streptomyces cattleya* and *Methanosarcina barkeri* showing that topologically, there is no need to use the other two Actinobacteria to produce both beta-lactam antibiotics. Two of them are presented in Fig. 2. The other two use another metabolite transport (*i.e.* L-2-aminoadipate) and are illustrated in the Supplementary Figure S5. In this case, the insertion of the reaction transforming 2-oxoadipate into L-2-aminoadipate is proposed in *M. barkeri* and L-2-aminodipate is transported into *S. cattleya.*

Three tentacular hyperacs are used in this case. One of the reactions is N-(5-amino-5-carboxypentanoyl)-L-cysteinyl-D-valine synthase that converts L-2-aminoadipate, L-valine and L-cysteine into *δ*-(L-2-aminoadipyl)-L-cysteinyl-D-valine, which is the starting point for the production of penicillin and cephalosporin C. All metabolites previously mentioned can be produced from pyruvate. The requirements to produce the three substrates of N-(5-amino-5-carboxypentanoyl)-L-cysteinyl-D-valine synthase using a solution of minimum weight therefore force to go back into the bacterium producing both amino-acids (L-valine and L-cysteine), in this example *S. cattleya*. The two other tentacular hyperarcs correspond to the reactions for citrate synthase (converts acetylCoA, H_2_0 and oxaloacetate into citrate and CoA) and AcetylCoA:2-oxoglutarate C-acetyltransferase (transforms 2-oxoglutarate and AcetylCoA into Homocitrate ((R)-2-hydroxybutane-1,2,4 tricarboxylate).

### Industrial biotechnology: Production of 1,3-propanediol and methane

The compound 1,3-propanediol (PDO) is of high interest in biotechnology since it is used as a building block in polymers^23^. Bizukojc *et al*.^10^ reported that the co-culture of the 1,3-propanediol producer *Clostridium butyricum* with a methanogenic Archaea, namely *Methanosarcina mazei*, could lead to a better yield of production. Indeed, in *C. butyricum*, production of PDO leads to the production of acetate as well as of a side-compound, the latter then participating in the production in *M. mazei* of methane, which is the main molecule in the composition of biogas.

In this example, another PDO producer and Enterobacteria glycerol scavenger, namely *Klebsiella pneumoniae*, is associated with *Methanosarcina mazei* to produce 1,3-propanediol and methane. Both organisms have the capacity to produce the target compounds. Hence, no reference organisms were used. The weights were first set as in the previous section (*i.e. w*_*worker*_ = 1, *w*_*other*_ = 100, *w*_*transition*_ = 100). The only authorised source was glycerol. Indeed, glycerol is a by-product of biodiesel biodiesel. It therefore is a substrate of choice for biotechnological processes^24^. In this case, we have two targets: 1,3-propanediol and methane.

**Figure 3:**
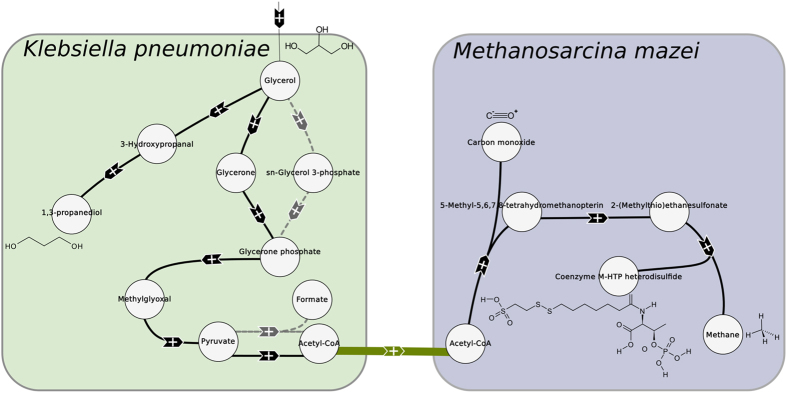
Solutions with uniform weights for the production of 1,3-propanediol from glycerol in *K. pneumoniae* and *M. mazei*. Black hyperarcs are endogenous reactions and green arcs represent transports. Grey dashed hyperarcs represent alternative paths.

We obtain six solutions with the same weight of 110 (1 transition and 10 endogenous reactions). In *K. pneumoniae*, there are two ways of reaching glycerone phosphate from glycerol. Moreover, two different reactions are possible to transform pyruvate into acetyl-CoA, one of them forming also formate. Finally, in the solutions obtained, there is also the possibility to exchange pyruvate instead of Acetyl-CoA. This therefore leads to six solutions (four of them are represented in Fig. 3, the last two are available in the Supplementary Figure S6).

In this case, the community does not exchange acetate but acetyl-CoA or pyruvate. In eukaryotes, transporters of acetyl-CoA are known in several pluricellular organisms and also in yeast. However, no transporter of acetyl-CoA has been detected in organisms close to the ones used in our case. Moreover, a pool of acetyl-CoA is essential to *K. pneumoniae*. Indeed, Jung *et al*.^25^ reported that a mutant with a reduced pool of acetyl-CoA showed growth retardation and redox imbalance. Therefore, it is not clear whether *K. pneumoniae* has an advantage in sharing acetyl-CoA or pyruvate (which is a substrate for the reactions producing acetyl-CoA). However, as stated previously, the production of 1,3-propanediol is associated with the synthesis of acetate and formate. Those by-products are inhibiting for *K. pneumoniae* and can reduce both its growth and the production of 1,3-propanediol^25^,^26^. Finally, *K. pneumoniae* possesses a citrate/acetate exchanger^27^ which is CitW, and *Methanosarcina* spp. can grow on acetate although other substrates might be preferred. This indicates the possibility of an exchange of acetate between the two organisms since transport is possible in both species. We therefore decided to diminish the weight of the transport of those organic acids to *w*_*transition*_ = 50.

**Figure 4:**
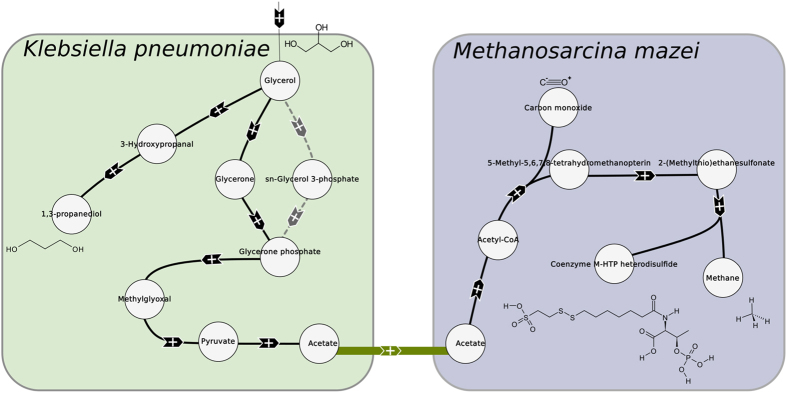
Solutions with reduced weight for acetate and formate to produce 1,3-propanediol from glycerol in *K. pneumoniae* and *M. mazei*. Black hyperarcs are endogenous reactions and green arcs represent transports, here acetate from *K. pneumoniae* to *M. mazei*. The grey dashed arcs represent the alternative solution.

Two minimum solutions were obtained with a weight of 61 (the acetate transport with *w*_*transition*_ = 50 and 11 endogenous reactions). They are presented in Fig. 4.

We can observe that this solution is really close to the previous one. Here, pyruvate is used to produce acetate (pyruvate:ubiquinone oxidoreductase) which is then exchanged from *K. pneumoniae* to *M. mazei*. The resulting pathway is in agreement with the one described by Sabra *et al*.^5^.

## Discussion

The method introduced in this paper allows to infer topological sub-networks to produce target compounds using one or several microorganisms forming a consortium. Ensuring that a component will be produced as much as it will be consumed according to stoichiometric coefficients leads to a more complex problem. Since we do not use such coefficients, a conservative hypothesis was adopted. This induces the exclusion of some cycles where a substrate used in a reaction is immediately formed again (such phenomenon appears for example in the phosphotransferase system in *E. coli*). Without stoichiometric coefficients, we cannot guarantee that the intermediate substrates of the cycles will be all regenerated by a solution. Prohibiting those cycles allows us to ensure that all solutions are feasible by themselves, meaning that all intermediates are at least as much produced as they are consumed (regardless of the remaining of the network).

Once a solution is obtained several points must be verified.

In the first example, only two of the four bacteria were selected to produce the two compounds of interest, showing the ability of our algorithm MultiPus to not only identify the less costly solution, but also to select the best consortium among a larger set of microorganisms given as input.

In this synthetic bacterial consortium defined by *Streptomyces cattleya* and *Methanosarcina barkeri*, pyruvate and either 2-oxoadipate or L-2-aminoadipate are exchanged between the two prokaryotes. The organisms therefore need to be able to export and uptake the three compounds. It was shown that *Methanosarcina barkeri*–the model species of the genus *Methanosarcina* whose properties are shared by most of the others^28^–grows on pyruvate, the uptake being done by passive diffusion^29^.

Moreover, *Streptomyces coelicolor* is able to transport monocarboxylates such as pyruvate by secondary carriers and active transporters^30^. Although pyruvate transporters have not yet been shown to exist in *S. cattleya*, it is probable that the transport of pyruvate is nevertheless possible since it happens in a closely related organism (*i.e. S. coelicolor*)^30^.

As concerns the second exchange, mitochondrial transporters for oxodicarboxylic acids (oxodicarboxylate carrier proteins (ODCs)) such as 2-oxoadipate and 2-oxoglutarate were reported in yeast (*Saccharomyces cerevisiae*) and in human^31^,^32^. Both human and yeast ODCs catalyse the transport of 2-oxoadipate and 2-oxoglutarate by a counter-exchange mechanism. Moreover, L-2-aminoadipate is also transported by the human ODC^31^. However, no homologous genes were found in Archaea and Actinobacteria (using a Blast analysis), neither did we find any information about the presence of such transporters in *Methanosarcina* or *Streptomyces*. Further experiments will therefore be needed to determine whether the two species constituting the microbial consortium do possess the ability to uptake and export 2-oxoadipate. Moreover, if it is confirmed that these two bacterial strains indeed lack this ability, an insertion of ODCs might still be possible, similarly to what was performed in *Escherichia coli* using human ODCs^31^.

Although the production of two beta-lactam antibiotics destroys the walls of positive Gram bacteria, *Streptomyces* is well-known for possessing a gene cluster which orchestrates antibiotic biosynthesis. Such cluster consists of resistance, transport and regulatory genes physically linked and coordinately regulated with genes encoding biosynthetic enzymes^33^. Among such species, *Streptomyces clavuligerus* produces several beta-lactam compounds, such as cephamycin C, clavulanic acid (an inhibitor of several beta-lactamases able to inactivate penicillins^20^) and other structurally related clavams^34^. Moreover, thienamycin, a carbapenem compound belonging to a class of beta-lactam antibiotics, is produced by *S. cattleya*. This metabolite employs a similar mode of action as penicillins through disrupting the cell wall synthesis (peptidoglycan biosynthesis) of various Gram-positive and Gram-negative bacteria. It further presents a resistance to bacterial beta-lactamases enzymes^20^,^35^. Therefore, *S. cattleya* could produce the two beta-lactam antibiotics without affecting its bacterial growth.

One must however call attention here to the fact that cultivating an aerobiose Actinobacteria and an anaerobiose Archaea in a same culture may be difficult. On one hand, several anaerobic-aerobic co-cultures have already been reported^36^. Indeed, because of the low solubility and diffusibility of oxygen in water, anaerobic micro-niches can be created and maintained in an aerobic environment^36^. On the other hand, we have here two mesophilic species: *Streptomyces* sp. (with a temperature growth interval between 25 °C and 35 °C) and *Methanosarcina* sp. (with an optimum of growth around 37 °C)^37^. In this context, the synthetic bacterial consortium will be able to grow together without major difficulties.

At their bacterial growth temperature (between 25 °C and 37 °C), we exclude a possible temperature-dependent biosynthetic pathway of antibiotic compounds as already reported for actinorhodin^38^. Indeed, the expression of the actinorhodin gene cluster was showed to be impossible at high temperatures (45 °C) and instead realised at 30 °C and at 37 °C, suggesting that it could thus depend on the temperature^38^. Under such conditions, the penicillin and cephalosporin C gene cluster should therefore be heterologously expressed by the consortium which should be able to produce the two well-known beta-lactam antibiotics.

In the second example, we retrieved a possible network for the joint production of 1,3-propanediol and methane. In Jung *et al*.^25^, attempts to reduce the production of by-products such as acetate through gene deletion led to a growth defect in *K. pneumoniae*. In those experiments, the yield of 2,3-butanediol (BDO) is improved by deletion of *pflB*, possibly because of the accumulation of pyruvate, a precursor of BDO. Indeed, *pflB* with *ldhA* encodes the pyruvate formate-lyase enzyme. Nevertheless in our case, pyruvate is not a precursor of PDO, hence the deletion of the same gene (*pflB*) would have a negative impact since the growth of the cells would be impaired by the redox imbalance created. Hence the possibility of the association with an acetogenic Archaea is of great interest to regulate acetate production.

In Bizukojc *et al*.^10^, an *in silico* simulation of the co-culture of another propanediol producer, namely *Clostridium butyricum*, with *M. mazei* showed an improvement in the growth of *C. butyricum* due to the consumption of acetate by *M. mazei*. Such consumption alleviates the inhibition of acetate. A similar effect should be expected for *Klebsiella pneumoniae*. The lighter weight assigned to the exchange of acetate allowed us to retrieve a feasible solution. Although acetate can be utilised almost completely by *M. mazei* for its growth, it is necessary to have methanol (present in raw glycerol obtained from biodiesel plant) in the medium to produce methane. However, even if the production of methane is low, the association of the two organisms will decrease the concentration of extracellular acetate, which is toxic for *K. pneumoniae*, hence increasing the yield of PDO. Co-cultures of *Clostridium* sp. associated to methanogenes such as *Methanosarcina sp.* CHTI55 have been described in the literature, showing acetate utilisation by methanogene organisms^39^. The use of an Enterobacteria, *Klebsiella pneumoniae*, as the propanediol producer in co-culture with methanogenes has been less described. Hence, more extensive tests on the feasibility using classical optimisation techniques are needed, even though the process and apparatus for such associations have been patented^40^.

As shown in this second application, we can assign a non uniform weight to the exchange of compounds between organisms, the insertion of exogenous reactions or the use of internal reactions. Using a biological *a priori* to tune the weights assigned to each reaction is helpful to obtain a realistic solution. Indeed, the weight of an inserted reaction can be set more precisely by taking into account, for example, gene-reaction associations. Reactions catalysed by protein complexes require the insertion of several genes, hence may be harder to handle than those associated to single genes. Using the AND/OR relations available in the SBML models, insertion weights may thus be adapted to reflect those difficulties. Moreover, if information about the inserted organisms is available, more complex weights can be computed, taking into account enzyme promiscuity, catalytic performance, gene compatibility^41^, but also for example the toxicity of side-products or even a known difficulty of enzyme incorporation. The exchange weights are harder to evaluate, however information about transporters (active or passive) for export and uptake may be taken into account to tune the exchange reactions. For example, a passive transporter is costless, molecules move across the membrane without energy input; on the contrary, an active transporter such as an ATP-powered pump will be costly since it requires the hydrolysis of ATP into ADP. Attributing a relative weight inside each category as briefly described above may be straightforward. What may be more difficult is to decide on how to balance such weights across the three categories. This may require some trial and error, and be dependent on the *in silico* experiment that is considered.

## Conclusion

We proposed a new topological method, called MultiPus, to select possible microbial consortia for the production of compounds of interest.

With MultiPus, any situation of both exogenous and endogenous compounds might be considered, as well as larger initial consortia whose final composition in terms of species is then optimised by the method. Finally, by setting the sources required, one can test the possibility of using low-cost substrates for the production of high value chemicals.

As a *post-processing* step, classical methods of flux balance analysis (using the inferred topological network) can be employed to predict product yield^42^,^43^,^44^. Gene over-expression and knock-out can moreover be explored in order to guarantee both growth and production of the compound(s) of interest, but also interaction among the species present in the consortium^45^,^46^.

Indeed, the species that are part of the consortium may not have the same growth rate, hence may not reach an equilibrium in terms of composition when all organisms are present. Stable growth and equilibrium in biomass of the community which is being considered is of importance, and stoichiometric models could be used to predict such equilibrium^11^,^47^. If balance cannot be reached, it is necessary to create a beneficial interaction among the organisms involved (mutualism or syntrophy) to guarantee the success of the synthetic community^48^. If needed, mutualism can be enforced by genetic engineering, for example by creating auxotrophic strains; this will force a cross-feeding between organisms, regulating the growth of the species composing the co-culture^49^,^50^.

This first model allows to infer topologically possible insertions for heterologous expression and the usage of a mixed culture for the production of exogenous and/or endogenous target compounds. Moreover, MultiPus may thus enable to establish which co-cultures could be interesting to use in order to avoid the inhibition of co-products (*e.g.* 1,3-propanediol). It is a good starting point, that should be associated in the future with more quantitative methods in order to guarantee maintenance and growth of the organisms in communities (for instance, taking into account account electron transport and/or red/ox balance).

The implementation of the algorithm is available at: http://multipus.gforge.inria.fr.

## Additional Information

**How to cite this article**: Julien-Laferrière, A. *et al*. A Combinatorial Algorithm for Microbial Consortia Synthetic Design. *Sci. Rep.* **6**, 29182; doi: 10.1038/srep29182 (2016).

## Supplementary Material

Supplementary Information
