# Supplementary material for: A Combinatorial Algorithm for Microbial Consortia Synthetic Design
Source: Sci Rep. 2016 Jul 4;6:29182. doi: 10.1038/srep29182 (PMC4931573; doi:10.1038/srep29182)
Supplement: Supplementary Information [file srep29182-s1.pdf]

A Combinatorial Algorithm for Microbial Consortia Synthetic Design (Supplementary Material)

Alice Julien-Laferrière, Laurent Bulteau, Delphine Parrot, Alberto Marchetti-Spaccamela,
Leen Stougie, Susana Vinga, Arnaud Mary, Marie-France Sagot

1 Complexity proofs

In Section 1 in the main text, we presented two complexity results whose proofs are given below.

Proposition 1. *The problem is W[1]-hard when parameterised by any combination of:* $|A'|$, weight(A'), $|T|$, $|S|$, *total number of tentacles of hyperarcs in* A'.

Proof. The reduction is from the Multi-Colour Clique (MCC) problem, defined as follows:

Input: A k-partite graph $G = (V, E)$ where $V = V_1 \cup V_2 \cup \ldots \cup V_k$.
Output: A k-clique of G, *i.e.* a set $V' = \{v_1, \ldots, v_k\}$, with $v_i \in V_i$ for all i, such that $\{v_i, v_j\} \in E$ for every pair $\{i, j\}$, $i \neq j$.

The MCC problem is W[1]-hard when parameterised by k.

Each set V_i is called a *(vertex-)class*. The set of edges can be partitioned into $\binom{k}{2}$ *edge-classes* $E_{i,j}$, according to the class of their endpoints (formally, $E_{i,j} = E \cap (V_i \times V_j)$).

Given an instance of MCC $G = (V, E)$, we build an instance of the Directed Steiner Hypertree problem as follows (see Supplementary Figure S1).

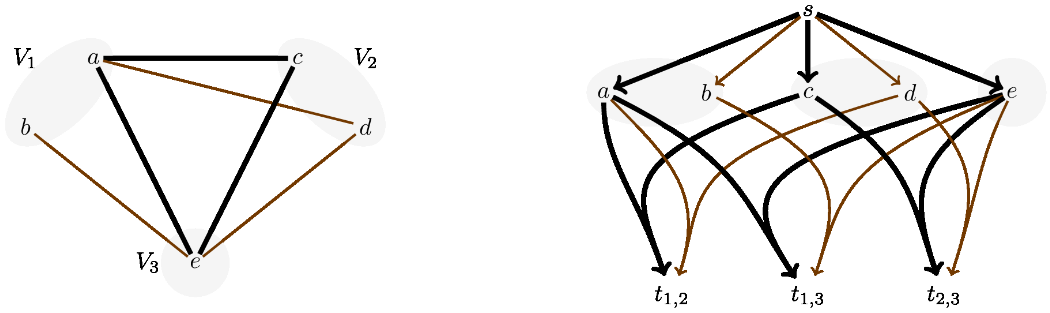

Supplementary Figure S1. Illustration of the parameterised reduction from Multi-Colour Clique (left) to Directed Steiner Hypertree (right) with 5 vertices and $k = 3$. A clique of the input graph and the corresponding optimal directed rooted hypergraph are depicted with bold edges.

Define a set of vertices W:

$$\begin{aligned} W = &\{s\} \\ &\cup \{x_v \mid v \in V\} \\ &\cup \{t_{i,j} \mid 1 \leq i < j \leq k\} \end{aligned}$$

Intuitively, this vertex set contains one source, one vertex for each original vertex of G, and one target for each edge-class. We write $S = \{s\}$ and $T = \{t_{i,j} \mid 1 \leq i < j \leq k\}$.

Define now a set of hyperarcs A, all having weight 1:

$$\begin{aligned} A = &\{a_v = (\{s\}, \{x_v\}) \mid v \in V\} \\ &\cup \{a_e = (\{x_u, x_v\}, \{t_{i,j}\}) \mid e \in E, e = \{u, v\} \in E_{i,j}\} \end{aligned}$$

This set contains two parts: first a set of simple arcs a_v allowing to reach any vertex x_v, then for each edge an hyperarc starting from both endpoints of the edge and reaching the target of the corresponding colour.

We now prove that $(\mathscr{H} = (W, A), S, T)$ admits a solution of weight at most $k + \binom{k}{2}$ if, and only if, G admits a k-clique. Note that this equivalence completes the parameterised reduction, since $|S|$, $|T|$, the solution size and its total number of tentacles are all bounded by a function of k.

If. Consider a k-clique K of G. Write E' for the set of $\binom{k}{2}$ edges used in K. It is easy to verify that $A' = \{a_v \mid v \in K\} \cup \{a_e \mid e \in E'\}$ is a valid solution: starting from the source s, each x_v, $v \in K$ can be reached using the corresponding arc $a_v \in A'$. Then for each $1 \le i < j \le k$, pick the edge $e = \{u, v\}$ having class $\{i, j\}$ in E': both vertices x_u and x_v have already been reached, hence the hyperarc a_e allows us to reach $t_{i,j}$. Overall, all targets have been reached with exactly $k + \binom{k}{2}$ hyperarcs.

Only if. Consider a solution A' of DSH for $(\mathscr{H}, S, T)$. Write $K = \{u \in V \mid a_u \in A'\}$ and $E' = \{e \in E \mid a_e \in A'\}$, that is, the set of vertices and edges of the original graph for which the corresponding hyperarc of $\mathscr{H}$ is used in A'. We will show that (K, E') forms a clique. The first observation is that $|K| + |E'| \le k + \binom{k}{2}$, since this is the maximum total weight of the solution.

For every $1 \le i < j \le k$, since $t_{i,j} \in T$, A' contains an hyperarc ending in $t_{i,j}$, so E' must contain some edge e having class $\{i, j\}$. This already shows that $|E'| \ge \binom{k}{2}$, which in turn yields $|K| \le k$.

Write $\{u, v\}$ for the endpoints of any $e \in E'$. Since x_u and x_v have in-degree 1 in $\mathscr{H}$, the arcs a_u and a_v must also belong to A', and $u, v \in K$. Hence all the endpoints of edges in E' are in K.

To sum up: E' is a set of at least $\binom{k}{2}$ edges with a total of only k endpoints: they are the edges of a clique of G.

□

Proposition 2. *The problem is NP-hard even when $|T| = 1$ and A contains only one tentacular hyperarc.*

Proof. The reduction is from the Directed Steiner Tree problem. Consider an instance of this problem, *i.e.* a directed graph $G = (V, A)$, a source $s \in V$, and a set of targets $T \subseteq V$. Create a directed hypergraph $\mathscr{H}$ from G by adding a vertex t and an hyperarc h from T to $\{t\}$. Then $(\mathscr{H}, \{s\}, \{t\})$ is an instance of the Directed Steiner Hypertree problem with a single target and a single tentacular hyperarc (see Supplementary Figure S2). Also, any solution necessarily takes the hyperarc h, and thus must cover its whole head, *i.e.* T. Hence the solutions of the Directed Steiner Hypertree problem exactly correspond to the solutions of the Directed Steiner Tree problem by adding h.

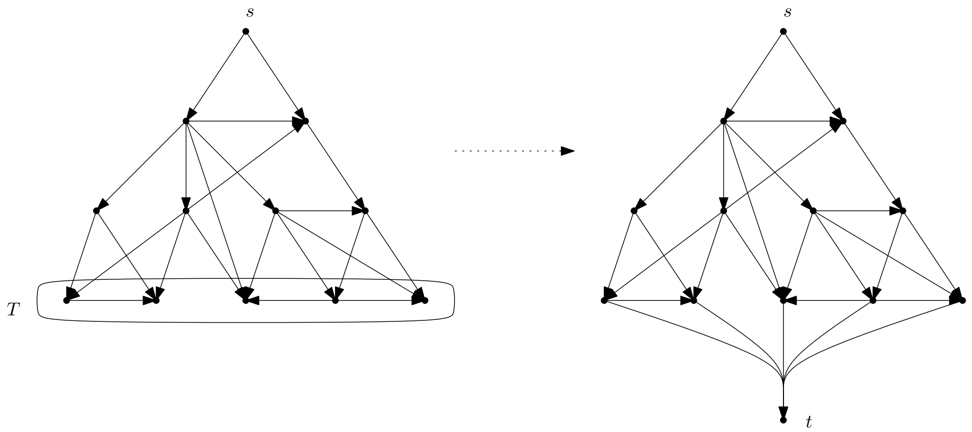

Supplementary Figure S2. Illustration of the reduction from Directed Steiner Tree (left) to Directed Steiner Hypertree (right), with a single tentacular hyperarc and a single target.

□

2 Graph Preprocessing

2.1 Metabolites removal

When creating the hypergraphs from the reconstructed metabolic networks, common cofactors and co-enzymes were removed. They were identified using the BRITE functional hierarchies of Kegg. The list of all filtered metabolites is given below:

- Adenosine 5'-triphosphate
- Nicotinamide adenine dinucleotide
- Nicotinamide adenine dinucleotide phosphate
- Coenzyme A
- Flavin adenine dinucleotide
- Pyridoxal phosphate
- S-Adenosyl-L-methionine
- UPD-glucose
- Heme
- Glutathione
- 3'-Phosphoadenylyl sulfate
- Riboflavin-5-phosphate

- Cytidine 5'-triphosphate
- Thiamin diphosphate
- Tetrahydrofolate
- Pyrroloquinoline quinone
- Coenzyme R
- Cobamide coenzyme
- 5-Dehydro-D-fructose
- Lipoate
- Methanofuran
- 5,6,7,8-Tetrahydromethanopterin
- 2-Mercaptoethanesulfonate (Coenzyme M)
- N-(7-Mercaptoheptanoyl)threonine O3-phosphate (Coenzyme B)
- Coenzyme F430
- Ubiquinone-10
- Heme A
- Heme O
- Molybdenum cofactor

This list has been made for general applications. Hence some of the metabolites may not be present in the networks used in the paper.

2.2 Graph transformation

The initial networks are obtained using the SBML file of the organisms. Each is modelled as a directed hypergraph (as described in the main text), $\mathscr{H} = (V, A)$.

Since we only want to take into account the hyperarcs of spreadness > 0 (*i.e.* such that $|\mathsf{src}(a)| > 1$), then all the hyperarcs of spreadness 0 (*i.e.* such that $|\mathsf{src}(a)| = 1$ and $|\mathsf{tgt}(a)| > 1$) are replaced by simple arcs in the network.

For all $a \in A$ such that $|\mathsf{src}(a)| = 1$ and $|\mathsf{tgt}(a)| > 1$, a vertex that will play the role of a pseudo-metabolite u_a is added to the network. The hyperarc selected is then removed and some arcs are added, one going from the tail vertex (substrate) to the pseudo-metabolite and the others from the pseudo-metabolite to the head vertices (products). To summarise, one hyperarc (the original one) is removed and $1 + |tgt(a)|$ simple arcs are added: the arc $(\mathsf{src}(a), u_a)$ and for each $v \in \mathsf{tgt}(a)$, an arc (u_a, v).

This step is not required when using the ASP solver.

2.3 Graph filtering

We call a reaction a a sink reaction if it has no product, hence is such that $|\mathsf{src}(a)| = 0$. Similarly, an import reaction a has no defined substrate ($|\mathsf{tgt}(a)| = 0$).

The filtering rules for the merged network are twofold:

1. Filtration of the sink and import reactions;

2. Filtration of the source and target metabolites that are not part of S and T.

The first step removes any arc (reaction) $a \in A$ such that $|\mathsf{tgt}(e)| = 0$ or $|\mathsf{src}(a)| = 0$.

The second step removes any vertex (metabolite) $v \in V$ such that $deg^+(v) = 0$ or $deg^-(v) = 0$. If $deg^+(v) = 0$, then all the outgoing reactions are removed. If $deg^-(v) = 0$, then the entering reactions e such that $v \in \mathsf{tgt}(e)$ (that is, which have v as product) are removed if $|src(a)| = |\mathsf{tgt}(a)| = 1$. Otherwise the reaction is simplified by removing v from its products ($\mathsf{tgt}(a) = \mathsf{tgt}(a) \setminus v$).

These two steps are repeated until the network is stable (no vertex or arc fits anymore the requirements of the filter).

In Supplementary Figure S3a, the hyperarcs of the type $S \to G + H$ will be divided into 3 arcs with the introduction of a node u'. The three created arcs are $(S, u'), (u', G), (u', H')$. The filtering step is applied twice. The first time, using rule 2, the vertices A, C, and F are removed, as are the reactions $A + S \to B$ and $E \to F$. The reaction $B \to C + D$ is simplified into $B \to D$. Using rule 1, the sink reaction of D ($D \to$) will be removed. The second time, according to rule 2, vertex E is deleted as is the reaction $S \to E$. The resulting network can be seen in Supplementary Figure S3b.

In Supplementary Figure S4, vertex I would be removed along with the two reactions $I \to$ and $F \to I$.

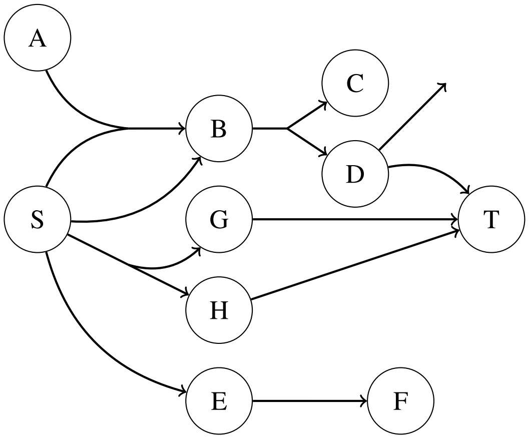

(a) Original network

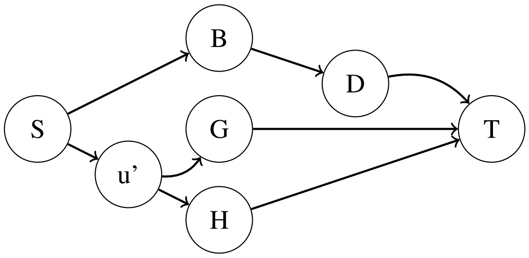

(b) Network after filtering. All arcs kept their weight, $S \rightarrow u'$ has weight w_{worker} but $u' \rightarrow G$ and $u' \rightarrow H$ will have a weight of 0.

Supplementary Figure S3. Toy example, before and after filtering. Here only one network is considered. All arcs have a weight of w_{worker} in S3a. In S3b, all arcs have a weight of w_{worker} except for $u' \rightarrow G$ and $u' \rightarrow H$ that have each a weight of 0.

2.4 Graph insertion

As introduced in the main text, once the networks of the members of the consortium (set O_w of the workers to be used to synthetically produce the compounds in T) are obtained, we add to each network the reactions taking place in the other organisms called reference (set O_o). A hyperarc is introduced if it is not present in the original network.

2.5 Transition

Transitions are added between all pairs of vertices that represent the same metabolites in different species with a weight of w_t.

2.6 Pseudo-sources and targets

We do not force the production of T in all organisms, one producer only is necessary. Hence we create pseudo-sources and pseudo-targets.

We connect every target $t_{i,j}$ corresponding to the metabolite i of an organism/network j to a pseudo-target t'_i by an arc $((t_{i,j}, t'_i)$ with a weight that is negligible compared to the other (regular) weights used (*e.g.* 10^{-6} was applied in the biological examples described in the main text). Reaching t'_i guarantees that at least one $t_{i,j}$ is reached. The same procedure is applied to the sources.

An example of the steps described above is depicted in Supplementary Figure S4.

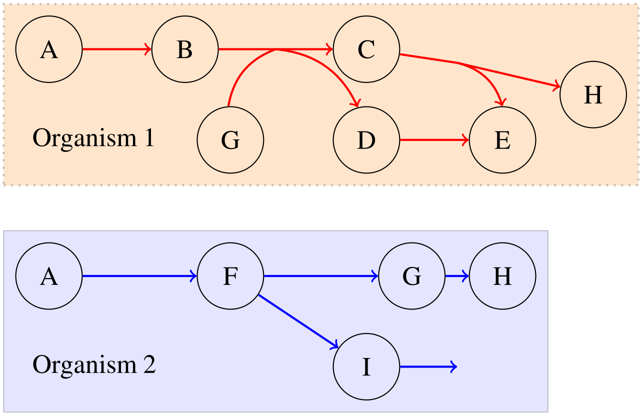

(a) Original networks, the two coloured squares represent two different organisms (red and blue).

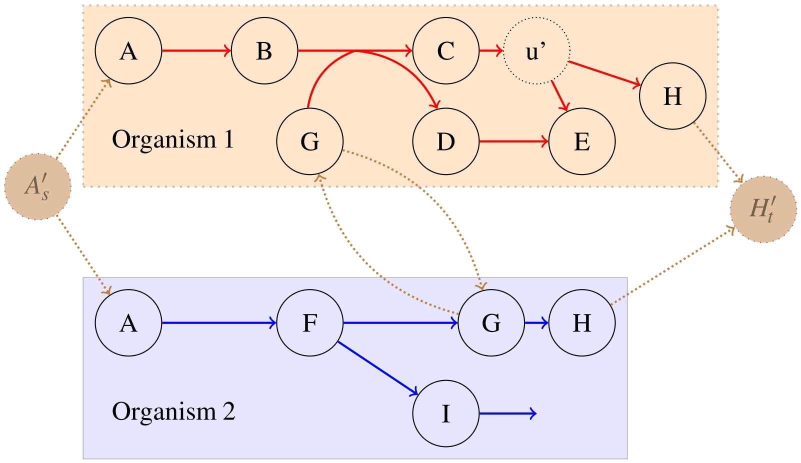

(b) Network after addition of the pseudo-source for A and pseudo target for H. Two transitions are added because both networks (organisms) share the vertex (metabolite) G. Moreover, the hyperarc $C \rightarrow E + H$ was decomposed as explained in Section 2.2

Supplementary Figure S4. Toy example for the transition reactions and addition of pseudo-sources and pseudo-targets.

3 Application: Alternative Results

We present here the minimum solutions that were not presented in the Figures of the main text.

3.1 Antibiotics production

For the antibiotics production, two alternative solutions are represented in Supplementary Figure S5.

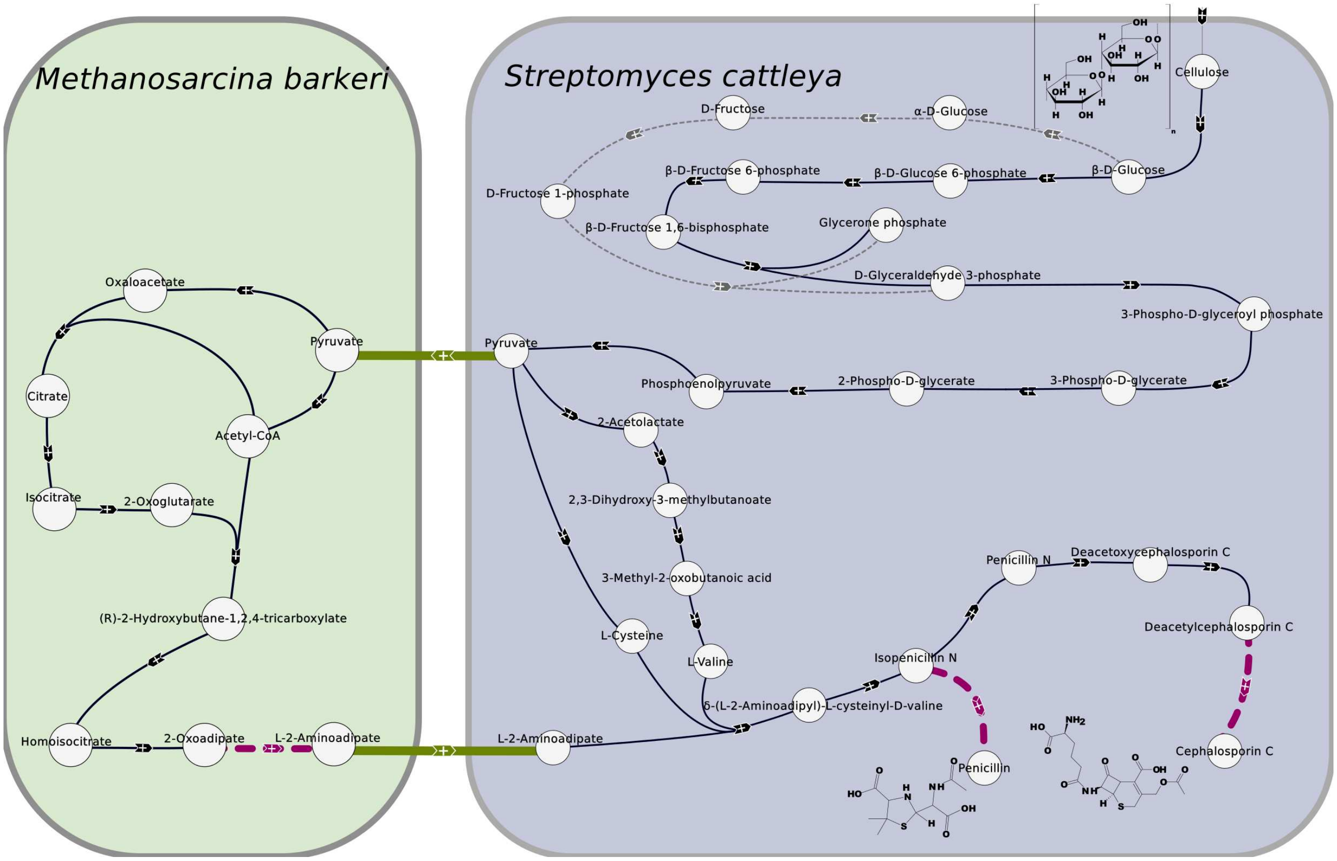

Supplementary Figure S5. Representation of two solutions of minimum weights. The circles are compounds. Black hyperarcs are endogenous reactions, that is reactions already present in the organisms forming the consortium, while purple-dashed hyperarcs are the reactions that were inserted. Green arcs represent the transport of pyruvate from *Streptomyces cattleya* to *Methanosarcina barkeri* and of L-2-aminoadipate from *M. barkeri* to *S. cattleya*. The widths of the arcs are proportional to the assigned weights. Grey dashed arcs represent an alternative path of endogenous reactions in the upper part of glycolysis. Hence, the second solution uses this path instead of the one just below to link β-D-glucose to D-glyceraldehyde 3-phosphate.

3.2 Industrial biotechnology: Production of 1,3-propanediol and methane

For the joint production of 1-3 propanediol and methane, two alternative solutions are represented in Supplementary Figure S6.

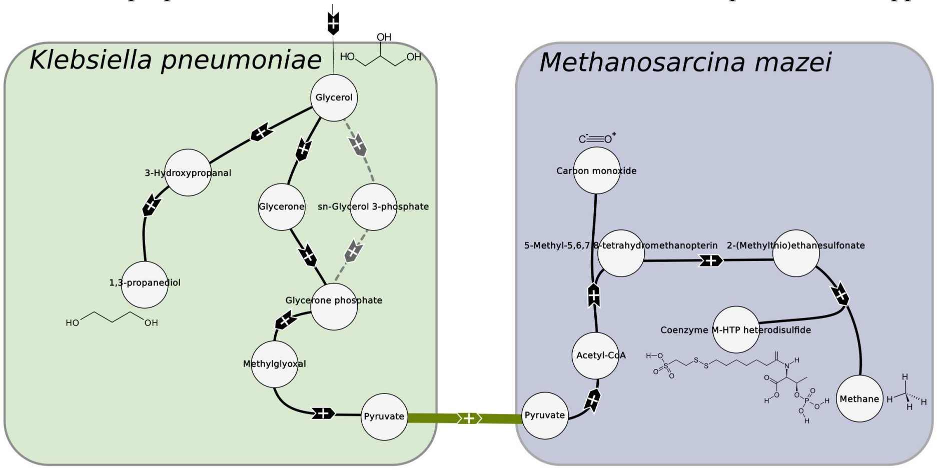

Supplementary Figure S6. The two others solutions in the case of uniform weights for the production of 1,3-propanediol from glycerol in *K. pneumoniae* and *M. mazei*. Black hyperarcs are endogenous reactions and green arcs represent transports. Grey dashed hyperarcs represent alternative paths.
